# Repulsive guidance molecule B inhibits metastasis and is associated with decreased mortality in non-small cell lung cancer

**DOI:** 10.18632/oncotarget.7463

**Published:** 2016-02-17

**Authors:** Jin Li, Lin Ye, Xiaoshun Shi, Jingyi Chen, Fenglan Feng, Yaoqi Chen, Yiren Xiao, Jianfei Shen, Peng Li, Wen G. Jiang, Jianxing He

**Affiliations:** ^1^ State Key Laboratory of Respiratory Disease, The First Affiliated Hospital of Guangzhou Medical University, National Clinical Research Center for Respiratory Disease, Guangzhou 510530, China; ^2^ Cardiff-China Medical Research Collaborative, Cardiff University School of Medicine, Cardiff, CF14 4XN, UK; ^3^ Key Laboratory of Regenerative Biology, South China Institute for Stem Cell Biology and Regenerative Medicine, Guangzhou Institutes of Biomedicine and Health, Chinese Academy of Sciences, Guangzhou 510530, China

**Keywords:** NSCLC, metastasis, RGMB

## Abstract

Repulsive guidance molecules (RGMs) are co-receptors of bone morphogenetic proteins (BMPs) and programmed death ligand 2 (PD-L2), and might be involved in lung and other cancers. We evaluated repulsive guidance molecule B (RGMB) expression in 165 non-small cell lung cancer (NSCLC) tumors and 22 normal lung tissue samples, and validated the results in an independent series of 131 samples. RGMB was downregulated in NSCLC (*P* ≤ 0.001), possibly through promoter hypermethylation. Reduced RGMB expression was observed in advanced-stage tumors (*P* = 0.017) and in tumors with vascular invasion (*P* < 0.01), and was significantly associated with poor overall survival (39 vs. 62 months, *P* < 0.001) and with disease-associated patient mortality (*P* = 0.015). RGMB knockdown promoted cell adhesion, invasion and migration, in both NSCLC cell lines and an *in vivo* mouse model, which enhanced metastatic potential. Conversely, RGMB overexpression and secretion suppressed cancer progression. The tumor-suppressing effect of RGMB was exerted through inhibition of the Smad1/5/8 pathway. Our results demonstrate that RGMB is an important inhibitor of NSCLC metastasis and that low RGMB expression is a novel predictor or a poor prognosis.

## INTRODUCTION

Lung cancer is one of the most commonly diagnosed malignancies and accounted for approximately 13% of the total cancer diagnoses in 2012. It is the leading cause of cancer death in males and second leading cause in females worldwide [1]. Non-small cell lung cancer (NSCLC) accounts for more than 85% of all lung cancer cases and includes adenocarcinoma, large-cell carcinoma, and squamous cell carcinoma [2]. Despite advances in early detection and improvements in treatment, the long-term survival rates for NSCLC remain poor as a consequence of distant metastasis.

Repulsive guidance molecules (RGMs) are glycosylphosphatidylinisotol (GPI)-linked membrane proteins consisting of three family members (RGMA, RGMB, and RGMC). Repulsive guidance molecule B (RGMB), also known as Dragon (‘turned on in DRG’), was the first RGM family member identified. RGMB, which is transcriptionally regulated by DRG11 [3], is a myelin-derived inhibitor of axon growth in the central nervous system [4] and a co-receptor for bone morphogenetic proteins (BMPs) [5].

BMPs are involved in the development and progression of various malignancies including breast, prostate, lung, and colorectal cancers, as well as melanoma and osteosarcoma [6, 7]. As a group of osteogenic factors, BMPs play key roles in the development of bone metastases [8–12] by coordinating bone formation, bone disruption, angiogenesis, and tumor growth through SMAD-dependent and -independent pathways [7]. RGMs may be involved in cancer development and progression by participating in the BMP signaling pathway. RGMB was also reported to be a receptor for PD-L2, a known ligand of PD-1. The PD-1/PD-L1 pathway provides a particularly promising strategy for cancer immunotherapy [13, 14]. Our previous study highlighted the aberrant expression profile of RGMs in breast cancer and indicated potential links between RGMs and patient prognosis [15]. However, the role of RGMB in lung cancer metastasis is unclear.

In the present study, we demonstrate that reduced RGMB gene expression is significantly associated with progression, metastasis, and poor prognosis in NSCLC. Suppression of RGMB promoted metastasis, thereby providing mechanistic insight into the role of RGMB in lung cancer development.

## RESULTS

### RGMB is downregulated in NSCLC tumors

**Figure 1:**
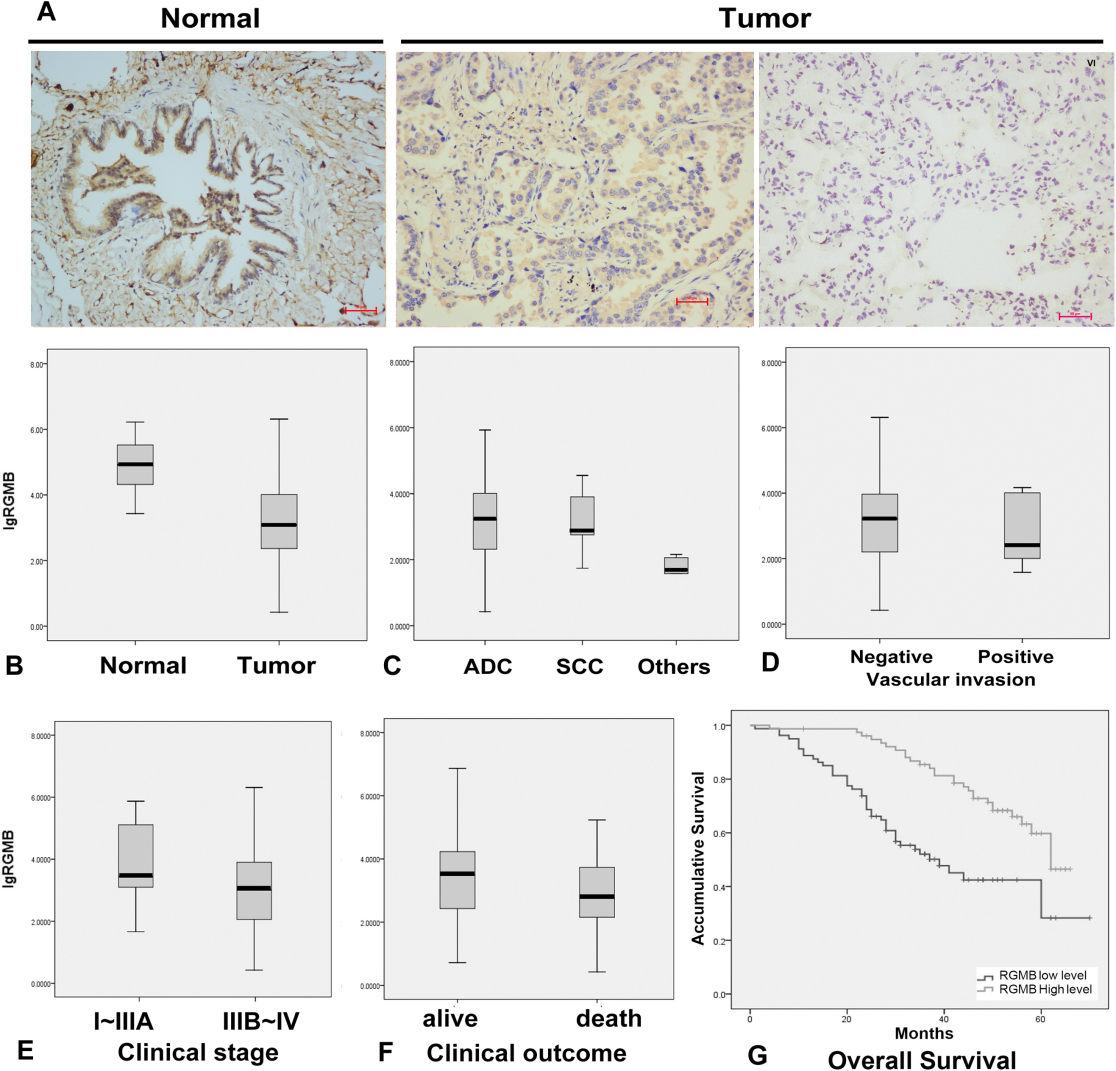
RGMB expression and association with lung cancer progression (**A**) Representative IHC images show a high level of RGMB expression in normal lung tissues (left, N), weak expression of RGMB in stage I tumors (middle), and the absence of RGMB expression in stage IV tumors (right) (scale bar = 100 μm). (B–E) RGMB transcription levels in (**B**) lung cancer [T, median: 1,217.815, interquartile range (IQR) 9,073.67, *n* = 296] and background tissues (N, median: 83,114.17, IQR: 259,640.2, *n* = 22, *P* ≤ 0.001), (**C**) in adenocarcinoma (ADC, median: 1,462.276, IQR: 10,349.5, *n* = 270), squamous cell carcinoma (SCC, median: 725.313, IQR: 10,651.07, *n* = 17) and other histological types (median: 48.912, IQR: 81.169, *n* = 9, *P* ≤ 0.001), (**D**) in patients with vascular invasion (median: 420.478, IQR: 7651.594, *n* = 8) and without vascular invasion (median: 1,319.333, IQR: 10814.95, *n* = 292, *P* ≤ 0.1), (**E**) in early-stage patients (median: 2,358.109, IQR: 109860, *n* = 261) and late-stage patients (median: 1,025.724, IQR: 9814.407, *n* = 33, *P* = 0.017). (**F**) Patients with better clinical outcomes have higher RGMB expression levels (median: 3,153.453, IQR: 17,853.13, *n* = 197) compared to those with poor outcomes (median: 710.577, IQR: 6,350.661, *n* = 85, *P* = 0.015). (**G**) Kaplan-Meier survival curves show the overall prognosis of patients with high RGMB expression (median: 62 months, 95% CI 58–67, *P* < 0.001) and low RGMB levels (median: 39 months, 95% CI 27–51) based on log-rank tests.

A statistically significant decrease in RGMB transcript expression (Figure 1B) was observed in NSCLC tumors compared to normal lung tissues. Immunohistochemical (IHC) staining of RGMB showed a cytoplasmic distribution in alveolar epithelial cells, bronchial epithelial cells, and parts of the lung cancer cells in human lung tissue sections (Figure 1A). In comparison with other NSCLC subtypes, the RGMB level was significantly higher in adenocarcinomas (*P* ≤ 0.001; Figure 1C). There were no significant differences in RGMB expression with respect to the age, sex, and smoking status of the patients.

### Reduced RGMB expression is associated with disease progression and poor prognosis

Lower RGMB transcript levels were observed in more advanced-stage tumors and tumors with vascular invasion (Figure 1D). Early-stage tumors had higher RGMB expression compared to more advanced-stage tumors (*P* = 0.017, Figure 1E). RGMB protein levels were reduced in advanced-stage tumors compared to early-stage tumors and normal tissues as assessed by IHC staining (Figure 1A).

The association between disease progression/prognosis and RGMB expression was initially examined in 165 NSCLC patients. Survival rates were compared between patient groups that were defined using the median RGMB expression level (3.12 copies) as the cut-off threshold. Patients with tumors expressing higher RGMB levels had longer overall survival (OS, median = 62 months; 95% confidence interval [CI], 58.9–66.1 months) compared to patients with tumors expressing lower RGMB levels (median = 39 months; 95% CI, 27.0–51.0 months) (Figure 1G; *P* < 0.001; Table 1). In addition, RGMB expression remained significantly correlated with OS after adjustments for age, sex, smoking status, and clinical stage, suggesting that RGMB is an independent prognostic predictor (Table 2). The association between RGMB expression and patient prognosis was then validated in a cohort of 131 NSCLC patients who were recruited between 2009 and 2010. Kaplan-Meier survival curves and log-rank tests showed a trend towards improved OS in patients with higher RGMB levels, though the improvement was not statistically significant.

**Table 1:** Log-rank test on OS for RGMB

	Group	Patients (No)	Median (Months)	95% CI	*P*
**Discovery set**	**Low RGMB**	80	39	27.0–51.0	4.07E-5
	**High RGMB**	77	62	58.9–66.1	
**Validation set**	**Low RGMB**	66	42	39.7–45.1	0.072
	**High RGMB**	58	59	52.4–65.4	

**Table 2:** Cox proportional hazard model

	Discovery			Replication			Fixed effect meta analysis			
**Factor**	HR	95% CI	*p*-value	HR	95% CI	*P*-value	HR	95% CI	*P*-value	*P*_het_
**RGMB**	0.57	0.458–0.710	4.85E–07	0.62	0.381–1.007	0.05	0.61	0.5–0.74	8.11E–07	0.19
**Age**	1.95	1.150–3.317	0.13	1.30	0.535–3.169	0.56	1.69	1.07–2.66	0.02	0.29
**Gender**	0.94	0.559–1.585	0.82	0.90	0.370–2.2.5	0.82	0.94	0.6–1.49	0.81	0.98
**Smoking status**	0.94	0.615–1.445	0.79	0.62	0.234–1.639	0.34	0.92	0.62–1.36	0.68	0.78
**Clinical stage**	1.61	1.269–2.041	8.87E–05	1.92	1.216–3.045	0.01	1.55	1.25–1.91	5.38E–05	0.47

Analysis of pooled data from the two cohorts using an inverse variance meta-analysis revealed a significant association between RGMB transcript levels and prognosis, which was independent of clinical stage (Hazard ratio [HR], 0.61; 95% CI, 0.5–0.74; *P* = 8.11 × 10^−7^) (Table 2). Because clinical stage is also a significant factor for prognosis, we further demonstrated the independent prognostic role of RGMB in early- and late-stage sub-groups of a cohort of lung cancer patients (Table 3). RGMB expression was found to be significantly associated with lung cancer-associated mortality (Figure 1F; *P* = 0.015).

**Table 3:** Subgroup analysis of Cox proportional hazard model

Subgroup	Factor	HR	LCI	HCI	*p*-value
**Early stage (I–IIIA)**	**RGMB**	0.2872	1.1794	1.6489	1.04E–05
	**age**	1.6655	2.6435	17.3451	6.33E–02
	**gender**	0.8284	1.6111	4.2156	5.04E–01
	**smoking**	0.7877	1.6454	3.4766	3.08E–01
**Late stage (IIIB–IV)**	**RGMB**	0.2736	1.0863	2.4699	3.36E–02
	**age**	1.6669	1.7580	137.7135	3.55E–01
	**gender**	1.0159	1.4215	18.8099	9.77E–01
	**smoking**	1.5842	2.3281	19.4845	1.51E–01

### RGMB suppresses adhesion, invasion, and migration of lung cancer cells *in vitro*

**Figure 2:**
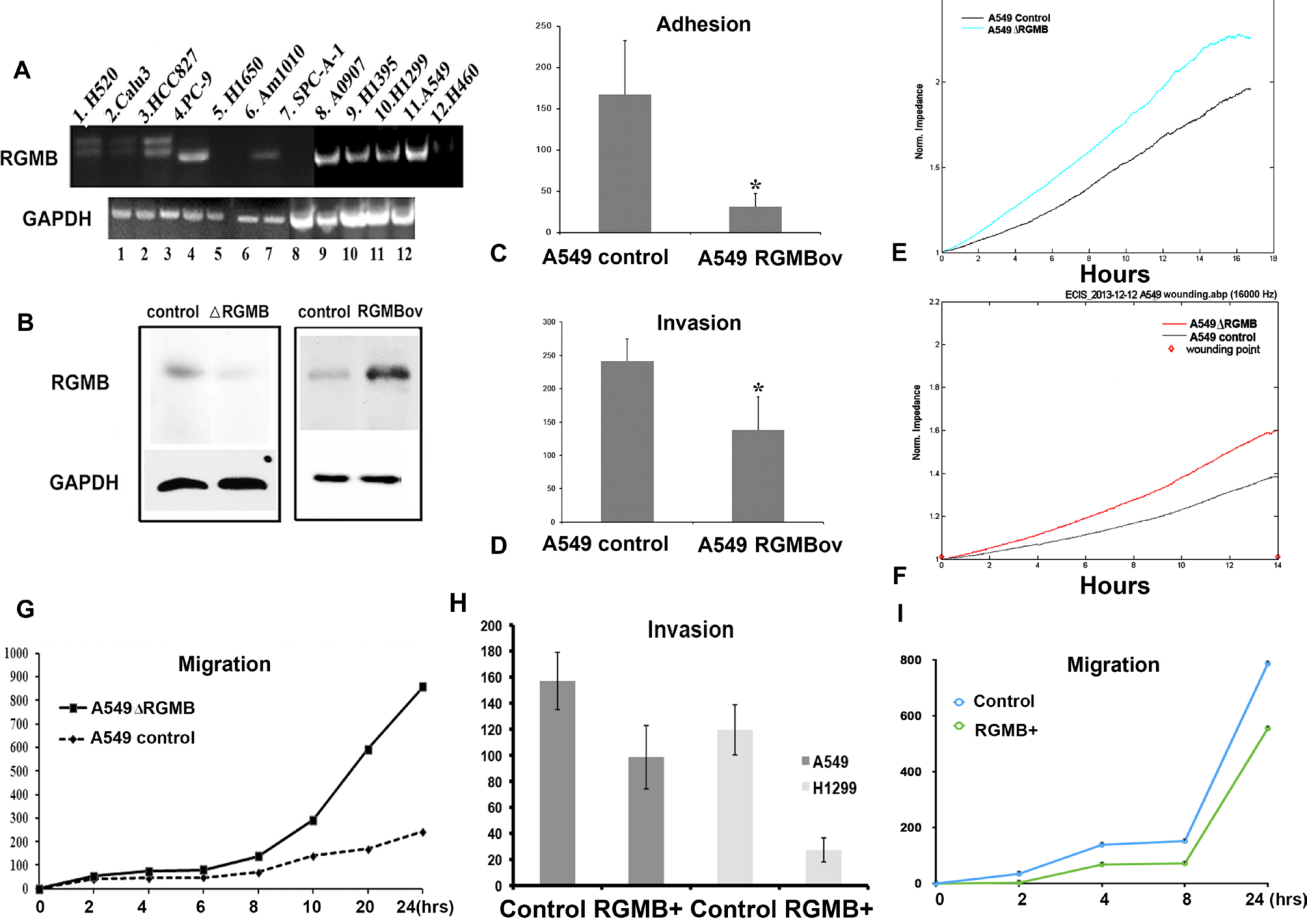
Manipulation of *RGMB* alters the malignant behavior of lung cancer cells *in vitro* (**A**) Screening of RGMB in lung cancer cell lines. (**B**) Western blot showing that RGMB expression is significantly repressed or increased in A-549 cells. RGMB can be detected in A-549 cells at a molecular weight of approximately 45 kD, with slightly weaker staining of another band observed at a lower molecular weight. The image is representative of three independent experiments. (**C**–**F**) RGMB overexpression inhibits cell adhesion (C) and invasion (D) in A-549 cells, *P < 0.05. ECIS assay showing that the repression of RGMB expression enhances the ability of lung cancer cells to adhere (E) and migrate (F). (**G**) Wound healing assay showing the enhanced migration ability of RGMB-knockdown A-549 cells. A comparison of the migration distance at each time point is shown in the graph. (**H**–**I**) Co-culture of RGMB-overexpressing cells with A-549 or H1299 cells inhibits the ability of the cancer cells to invade (H) and migrate (I) compared to co-culture with vector control cells.

The association between RGMB expression and NSCLC prognosis prompted us to investigate whether manipulation of *RGMB* gene expression would alter the malignant phenotype. We first evaluated *RGMB* expression in lung cancer cell lines (Figure 2A). RGMB expression was observed in PC-9, A0907, H1395, H1299, and A-549 cell lines and was weakly expressed in H520, HCC827 and Am1010 cell lines. Therefore, RGMB was knocked down or overexpressed in both A-549 and H1299 cells (Figure 2B, Figure S1).

Although alteration of RGMB expression did not impact the proliferation of NSCLC cells (Figure S2A, S2B and S2C), cell adhesion and invasion were significantly enhanced or suppressed by RGMB knockdown or overexpression, respectively (Figure S3, Figure 2C and 2D). Electric cell substrate impedance sensing (ECIS) and wounding assays showed that the ability of the lung cancer cells to attach (Figure 2E) and migrate (Figure 2F, 2G and Figure S3) was enhanced by RGMB knockdown. An Fc fusion protein variant of RGMB that is released from cells in a soluble form was reported previously [3]. In the present study, invasion and migration assays showed that co-culture of RGMB-overexpressing cells (using a transwell system) with lung cancer cells resulted in reduced invasion and migration of the lung cancer cells. It is possible that this effect was mediated by the RGMB Fc protein (without the GPI anchor) (Figure 2H and 2I).

### RGMB influences the metastatic dissemination of lung cancer cells in a murine tumor model

**Figure 3:**
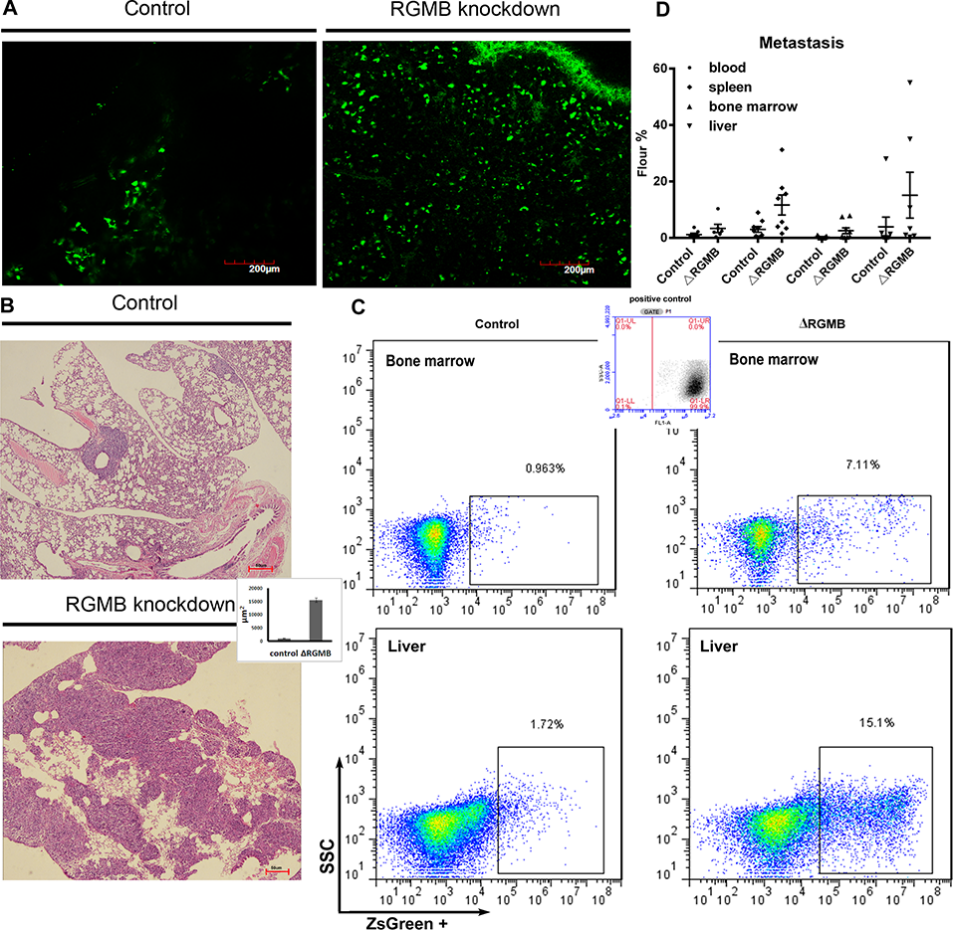
RGMB gene manipulation alters lung cancer metastasis *in vivo* (**A**) Repression of RGMB increases migration of tumor cells into the lung. GFP-labeled tumor cells observed with a two-photon microscope are shown (scale bar = 200 μm). (**B**) Representative image showing metastatic tumor loci that formed in the lungs of mice injected with RGMB-knockdown or vector control A-549 cells. The bar graph shows counting of the tumor loci size using Image J in three fields per slide. (**C**) Metastatic lung cancer cells detected by flow cytometry. (**D**) Detection of metastatic RGMB-knockdown cells in different organs compared to control cells.

To examine the role of RGMB in tumorigenicity *in vivo*, we established xenograft models by subcutaneously injecting A-549 RGMB-knockdown cells into mice. Metastatic dissemination of the tumor cells to the lungs was observed by two-photon microscopy. No differences were observed in primary tumor growth between control and RGMB-knockdown A-549 subcutaneous tumors (Figure S2D). However, an increased number of GFP-labeled RGMB-knockdown cells migrated to the lung compared to the A-549 empty vector controls (Figure. 3A). Moreover, the metastatic lung tumor burden significantly differed between the vector and RGMB-knockdown groups 6 weeks after tumor cell inoculation (Figure. 3B). More cancer cells were present in the circulation and at distant metastatic sites such as the liver, bone marrow, and spleen following RGMB knockdown as detected via fluorescence activated cell sorting (FACS) (Figure 3C and 3D; Table 4).

**Table 4:** The in vivo metastasis of cancer cells

Cell line/metastasis site	Cancer cell rate (percentage, mean ± SD)			
	Blood	Spleen	Bone marrow	Liver
control	1.18 ± 0.15	3.02 ± 1.46	0.29 ± 0.12	3.93 ± 0.05
RGMB knockdown	3.38 ± 0.88	11.66 ± 13.87	2.56 ± 0.20	15.14 ± 4.80
*P* value	0.15	0.02	0.04	0.02

### RGMB signaling model

**Figure 4:**
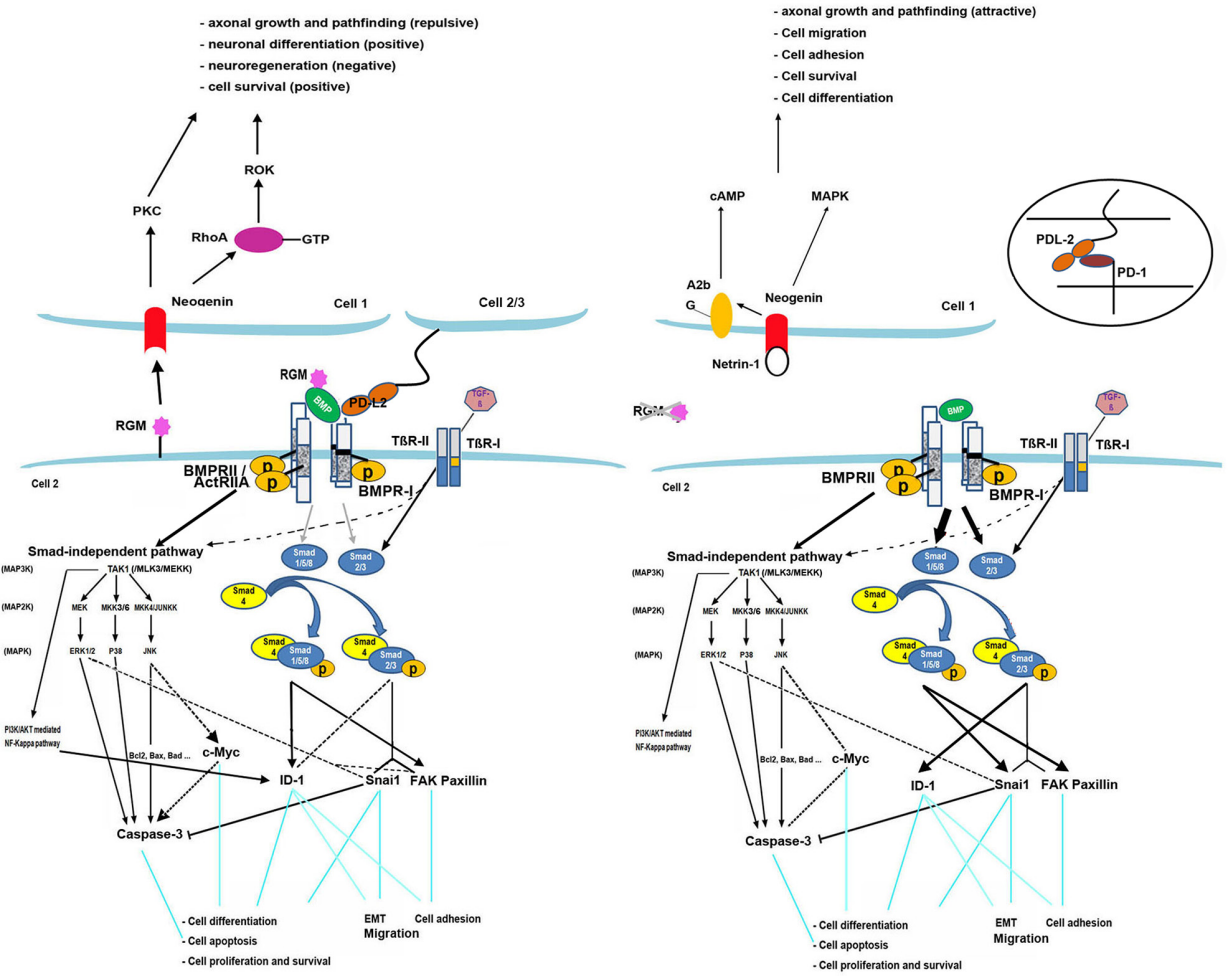
RGMB model Knockdown of RGMs facilitates BMP signaling through the Smad1/5/8 pathway and partly through Smad2/3. The MAPK-JNK pathway is inhibited thereby mediating the expression of BMP downstream genes. FAK/paxillin is induced by BMP through Smad1/5/8 and TGF-β through Smad2/3. It also interacts with components of the ERK1/2 pathway [26]. Expression of focal adhesion proteins is primarily stimulated by activation of Smad1/5/8 and partially by Smad2/3 in the absence of RGMB, which leads to increased cell adhesion in breast cancer. Snai1 can be induced by TGF-β through Smad2/3 [27]. Regulation of Snai1 is also associated with Smad1/5/8 activation after RGMB knockdown, which induces EMT. ID-1 is upregulated or downregulated by TGF-β via Smad2/3, depending on the phase [28, 29], and it can also be activated by BMPs through Smad1/5/8 and the PI3K/Akt pathway [30–32]. Knockdown of RGMs in prostate cancer cells has been shown to result in upregulation of ID-1 primarily through Smad2/3, which may promote cell proliferation [33], migration [34], and adhesion [32]. Knockdown of RGMB suppressed expression and activation of caspase-3 (executes the cell apoptotic program) and reduced c-Myc expression, which may also be involved in the regulation of cell death. Binding of RGMs to neogenin may trigger different pathways that mediate neuronal growth, differentiation, and iron metabolism [35–38]. RGMB binds PD-L2, which promotes respiratory immunity independent of another PD-L2 receptor, PD-1, which inhibits antitumor immunity [14].

**Figure 5:**
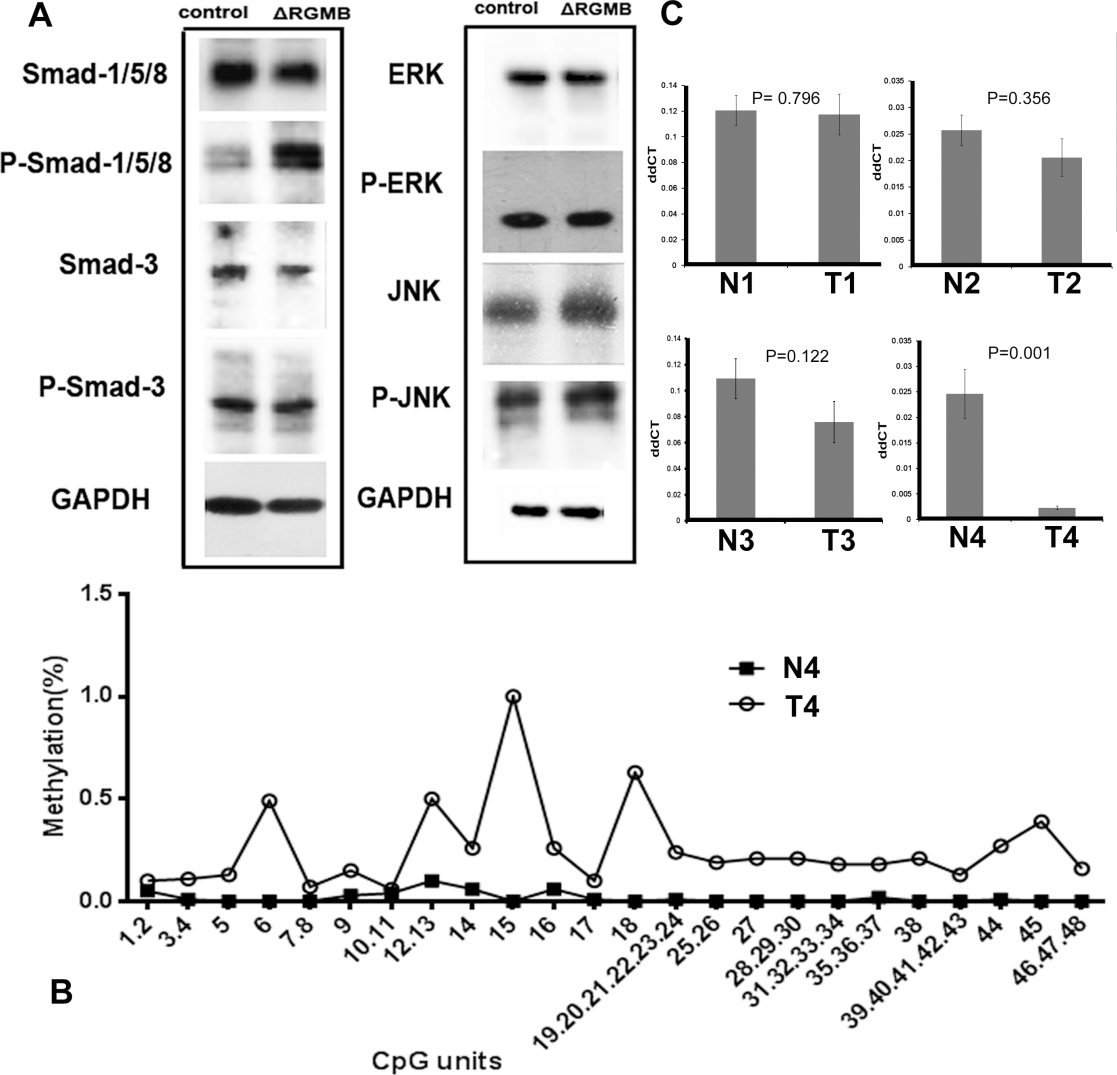
The signaling pathway and potential mechanism of RGMB downregulation in lung cancer (**A**) Western blot results showing that RGMB knockdown induces Smad-1 phosphorylation (P-Smad-1) but has no stimulatory effect on the expression (ERK, JNK) or phosphorylation (P-ERK, P-JNK) of components of the Smad-independent pathway. (**B**) Methylation levels of *RGMB* CpG units in N4 and T4 samples. (**C**) RGMB expression in tumor and normal samples, as determined by qPCR assays.

Based on previous studies and our findings, the model for RGMB signaling we propose is described in Figure 4. There are two signaling pathways by which BMPs activate intracellular signaling molecules: the Smad-dependent and Smad-independent pathways [16]. Smad1/5/8 and Smad2/3 are responsible for the Smad-dependent pathway. The Smad-independent (MAPK) pathway consists of a chain of activation in which MAPK (as JNK, ERK1/2) is activated following sequential activation of MAP4K/MAP3K/MAP2K [17]. Two different types of serine-threonine kinase transmembrane BMP receptors (Types I and II) are indispensable for both pathways upon activation by BMPs [18]. An increase in phosphorylated Smad-1/5/8 levels was observed in RGMB knockdown cells (Figure 5A). In addition, knockdown of Smad1 eliminated RGMB knockdown-enhanced cell migration (Figure. S4). No changes were observed in activation of ERK and JNK in RGMB knockdown cells (Figure 5A).

### Potential mechanism of RGMB downregulation in lung cancer

To investigate the potential mechanisms underlying downregulation of RGMB in lung cancer, the methylation of the *RGMB* gene was screened in four paired human samples. In total, 83 CpG positions derived from 67 CpG units in RGMB were analyzed. Of these positions, successful measurements for all four samples were obtained for 72% of the CpG units. We found that most of the CpG units in three tumor samples (T1, T2, and T3) were methylated to a very low extent, with an average methylation level of < 20%. This was similar to the level of methylation in the normal samples. Only one sample (T4) had four CpG units that displayed methylation levels > 50%, which differed from the normal tissue (low methylation level) (Figure 5B).

We performed real-time quantitative PCR (qPCR) assays to further investigate the association between CpG unit methylation and RGMB expression. The tumor (T4) that had a higher methylation level compared to that of the normal adjacent tissue (N4) had significantly decreased RGMB expression (Figure 5C). However, the other three tumors (T1, T2, and T3) did not show obvious changes in methylation status. These results suggest that the methylation of CpG units in the RGMB promoter region is only one of the mechanisms by which RGMB is downregulated in some lung cancer tumors.

## DISCUSSION

The present study revealed that RGMB is downregulated in lung cancer and that downregulation of RGMB is correlated with poor prognosis and shorter OS. Our results also suggest that hypermethylation of the *RGMB* promoter is one mechanism underlying the downregulation of RGMB in lung cancer. We performed an analysis of 448 lung adenocarcinomas (LUAD) and 364 lung squamous cell carcinoma (LUSC) methylation normalized RNAseq2 datasets, which were downloaded from The Cancer Genome Atlas Research Network (http://cancergenome.nih.gov/). The methylation and log-normalized RNAseq2 data demonstrated an inversely proportional relationship in both the LUAD and LUSC datasets (Figure S5). These results indicate that RGMB expression is downregulated as a result of the degree of methylation, which is in line with our observations. Furthermore, there is evidence that *RGMB* gene expression could be up-regulated by hepatocyte growth factor in HECV endothelial cells [19]. RGMB is expressed by different cell types and may contribute to tumor growth and progression. Additionally, it may be regulated by a variety of cytokines/pathways that are yet to be fully investigated.

In line with the observations from the NSCLC tumors, our *in vitro* and *in vivo* data have shown that RGMB can inhibit NSCLC cell metastasis. Further investigations have revealed that the inhibitory effect of RGMB in lung cancer is occurs primarily through suppression of the Smad-1/5/8-dependent pathway. The activation of Smad-1/5/8 observed in RGMB knockdown cells could have resulted in transcriptional regulation of BMP-responsive genes [20, 21], and enhanced invasion and migration of cancer cells. Blocking Smad1 in RGMB-knockdown A-549 cells diminishes the RGMB knockdown-enhanced migration of these cells suggesting that RGMB can inhibit the metastatic potential of lung cancer cells through suppression of the Smad-1/5/8 pathway.

In summary, our results suggest that the RGMB is a prognostic biomarker in NSCLC and has therapeutic potential because it can prevent metastasis.

## MATERIALS AND METHODS

### NSCLC tumor samples

All samples were collected during surgery at the First Affiliated Hospital of Guangzhou Medical University. In total, 165 tumor samples and 22 normal lung tissue samples were obtained from patients who were enrolled in the study between April 2007 and June 2009. An additional 131 tumor samples were collected from patients between June 2009 and June 2010. The tissues obtained during surgery were snap-frozen in liquid nitrogen and stored at −80°C. The diagnosis of lung cancer was confirmed immediately after surgery, and the presence of tumor cells was verified by a pathologist (GHL) using frozen sections stained with hematoxylin and eosin, in accordance with the World Health Organization guidelines [22]. The tumor stage was assigned according to the American Joint Committee for Cancer criteria [23]. Background tissues were confirmed to be free of tumor deposits. The follow-up time was up to 60 months. All human specimens and data were obtained according to a protocol reviewed and approved by the local ethical committee, and all patients signed an informed consent form. Detailed demographic information for all of the patients involved in this study is shown in Supplementary Table 1.

### RGMB expression analysis by real-time qPCR and immunochemical staining

Real-time qPCR was performed using a StepOne Plus real-time PCR system (Applied Biosystems, Foster City, CA, USA). Briefly, RNA was isolated using the TRI Reagent from Sigma (USA) according to the manufacturer's instructions. RNA (0.5 μg) was reverse transcribed into cDNA using the iScript^™^ cDNA Synthesis kit (Bio-Rad Life Science, USA). The qPCR assay was based on Amplifluor technology. The following primers were designed using the Beacon Designer software and included a Z sequence: RGMB- forward, 5′-GGCCT GGCCACTCATAGATA-3′, reverse,5′-ACTGAACCTGACCGTACATCATCTGTCACAGCTTGGTA-3′;GAP DH- forward, 5′-AAGGTCATCCATGACAACTT-3′, reverse, 5′-ACTGAACCTGACCGTACAGCCATCCAC AGTCTTCTG-3′. The qPCR assay was performed with an ABI Prism 7900 System (Applied Biosystems) using the Absolute Q-PCR ROX Mix (Thermo Fisher Scientific, Waltham, MA, USA). The transcript copy number was calculated based on an internal standard, GAPDH, according to a previously reported method [24]. All of the assays were performed in triplicate. RGMB expression levels were dichotomized according to the median values. The tissue blocks were cut into 5-μm sections and processed for IHC in accordance with a previously described protocol [15].

### Statistical analysis

Progression-free survival (PFS) was defined as the minimum interval from the date of diagnosis to the date of tumor recurrence, progression, occurrence of a second malignancy, death, or the last follow-up. OS was defined as the interval from the date of diagnosis to the date of death or the last follow-up. Living patients with local recurrence or metastasis were considered to be ‘in disease survival’. Correlations between RGMB levels and other demographical and clinical features were assessed using Mann-Whitney rank sum and Kruskal-Wallis one-way analysis of variance tests.

Patients were assigned to two equal-sized groups defined by median RGMB expression. Kaplan-Meier survival curves and log-rank tests were used to evaluate differences in OS and PFS between the two groups. The Cox proportional hazards model was used to estimate hazard ratios (HRs) and 95% CIs for RGMB levels with consideration of other demographic and clinical parameters. The survival analyses were performed using the SPSS statistical software (Version 11; SPSS, Chicago, IL, USA). All of the statistical tests were two-sided, and *P* < 0.05 was considered statistically significant.

### Cell lines, antibodies, and reagents

Human lung adenocarcinoma cell lines A-549 and H1299 were obtained from the American Type Culture Collection (Manassas, VA, USA). Antibodies against the following proteins were used: GAPDH, RGMB, Smad-1/5/8, Smad-2/3, JNK, ERK1/2 (Santa Cruz Biotechnology, Santa Cruz, CA, USA), P-Smad-1/5/8, P-Smad-3, P-JNK, and P-ERK1/2 (Cell Signaling Technology, Danvers, MA, USA).

### Functional studies

For wound healing assays, cells were seeded at a density of 1 × 10^6^ cells/well into 6-well plates and then pre-incubated for 24 h before a wound was created in the cell monolayer using a plastic tip. The cells were then grown in culture medium supplemented with 1% fetal bovine serum. The migration of the cells was tracked and recorded for 24 h using a microscope (Leica DFC450). Cell migration was determined by measuring distances between parallel lines indicating the initial sites and migratory sites. The procedure was repeated three times. Cell invasion assays were performed using modified Boyden chambers (8 μm) with polycarbonate membranes (Corning, Corning, NY, USA) coated with 500 μg/mL Matrigel (BD Biosciences, San Jose, CA, USA). Cells (20,000) were added to transwell inserts on top of the artificial basement membranes. The rate of cell growth was measured using a MTS proliferation assay (Cell Titer 96 Aqueous One Solution Cell Proliferation Assay; Promega, Madison, WI, USA), which was performed according to the manufacturer's instructions.

### Electric cell substrate impedance sensing (ECIS) assay

An ECIS Z model (Applied Biophysics Inc., Troy, NY, USA) was used to analyze migration in a wound model [25] in which 8W10E arrays were used. Each of the eight wells contained 10 circular 250-μm diameter active electrodes connected in parallel on a common gold pad. Each well had a substrate area of 0.8 cm^2^ and a maximum volume of 600 μL. In a confluent cell layer, an average of approximately 500–1,000 cells were measured by the electrodes.

Following the treatment of the array surface with a cysteine solution (10 mM), the arrays were incubated in serum-free medium for 1 h. The same number of cells (80,000/well) was added to each well. When the cells reached confluence, the monolayer was electrically wounded at 4 V and 30 kHz for 10 seconds. The *in vitro* migration rate was determined using a previously described method [19].

### Western blot analysis

Protein extraction was performed using RIPA buffer [50 mM Tris, 150 mM NaCl, 1% Triton X-100, 0.1% SDS, and 1% sodium deoxycholate (pH 7.4)] supplemented with protease inhibitors (phenylmethylsulfonyl fluoride and PImix). Protein concentrations were then measured using a Bio-Rad protein assay kit (Bio-Rad, Hercules, CA, USA). Protein lysates were resolved via SDS-PAGE and then transferred to nitrocellulose membranes (Hybond^™^-P; Amersham Biosciences, Piscataway, NJ, USA), which were blocked with TBS containing 0.2% Tween-20 (TBST) and 5% non-fat dry milk. The membranes were incubated with primary antibodies overnight at 4°C. An antibody against GAPDH was used as a control for protein loading. After washing with TBST, the membranes were incubated with a secondary antibody for 1 h at room temperature before exposure.

### RGMB overexpression and knockdown

Full-length *RGMB* was synthesized, sequenced, and cloned into the pLVX-mCMV-ZsGreen-puro plasmid (Biowit Technologies Ltd., China). For overexpression experiments, A-549 cells were transfected with 10 μg of DNA and 20 μL of Lipofectamine 2000 (Invitrogen, Camarillo, CA, USA). The transfected cells were selected in complete medium containing puromycin (Invitrogen) at 5 μg/mL. To knock down RGMB expression, a shRNA that specifically targeted human *RGMB* (sequence: 5′-UUAUUAUCUAUGAGUGGCCAGGCCC-3′) was purchased from Life Technologies (validation information for this shRNA target sequence can also be obtained from Sigma-Aldrich: product number: TRCN0000365323). Knockdown was validated using qPCR and Western blotting. RNAi expression vectors (pLVX-ShRNA-Puro-RGMB and the control plasmid pLVX-ShRNA-Puro-hScramble) were prepared using T4 DNA ligase and the *EcoR*I and *Mlu*I restriction endonucleases (New England Biolabs, Ipswich, MA, USA) and were isolated using a plasmid isolation kit (Qiagen, Valencia, CA, USA) according to the manufacturer's protocol. Lentiviral particles were produced via the transient transfection of 293FT cells (Invitrogen) using the Lipofectamine 2000 reagent (Invitrogen). For establishment of cell lines with the stable knockdown and overexpression of RGMB, cells were selected in culture medium containing 5 μg/mL puromycin (Invitrogen) after transfection. After 6 weeks of selection, resistant colonies stably transfected with pLVX-mCMV-ZsGreen-puro-RGMB or pLVX-ShRNA-Puro-RGMB, or the corresponding controls were pooled.

### Animal studies

Animal experiments were performed in the Laboratory Animal Center of Guangzhou Institutes of Biomedicine and Health (GIBH), and all animal procedures were approved by the Animal Welfare Committee of GIBH. NOD-*scid*-*IL2Rg*−/− (NSI) mice were derived at the GIBH. All mice were maintained in Specific pathogen-free cages and provided with autoclaved food and water. Protocols were approved by the relevant Institutional Animal Care and Use Committee. Tumorigenicity was investigated with tumor xenograft experiments. Briefly, female 8–10-week-old NSI mice were injected subcutaneously with 100 μL of a suspension of RGMB knockdown cells or vector control A-549 cells (approximately 1 × 10^6^ cells). Mice injected with saline were used as sham controls. Tumor formation was monitored for approximately 6 weeks and the tumor weight to body weight ratio calculated. Mice with a tumor size > 1 cm in any dimension were terminated. Tumor size was calculated according to the following formula: 0.5234 × [long diameter (short diameter)^2^]. To develop the metastatic model, 6–8-week-old female NSI mice were transplanted with 1 × 10^6^ A-549 cells through the fundus vein under sterile conditions. All of the mice were sacrificed by perfusion 6 weeks after the injection. The lungs were examined using a two-photon microscope (OLYMPUS, FV1200MPE) for the presence of ZsGreen-labeled metastatic cells. Specimens were then collected, fixed, and sectioned (5 μm) in preparation for hematoxylin and eosin staining to determine the site of metastasis. Peripheral blood monocytic cells, splenocytes, liver, and bone marrow cells were analyzed using an Accuri^™^ C6 system (BD Biosciences) for the presence of ZsGreen-positive lung cancer cells. Tumor loci and tumor cells were counted.

### Detection of *RGMB* CpG island methylation

Genomic DNA was extracted from four lung cancer samples and paired normal adjacent tissues using a QIAamp DNA Minikit (Qiagen). Genomic DNA (1 μg) was modified with sodium bisulfite using the EZ DNA methylation kit (Qiagen), and the DNA methylation levels in the clinical samples were determined using the MassARRAY platform (Sequenom, San Diego, CA, USA). Briefly, three fragments covering the *RGMB* promoter CpG islands were amplified from the bisulfite-modified DNA and 61 CpG sites were successfully tested. The following primers were designed by the Sequenom EpiDesigner:

5′-AGGAAGAGAGTTATTTTTATTTGAGGTT GATTGGG-3′ (sense) and 5′-CAGTAATACGACTCACT ATAGGGAGAAGGCTCCACAAACATTCCCTTATCT ACAAC-3′ (antisense); 5′-AGGAAGAGAGGTTGTAG ATAAGGGAATGTTTGTGG-3′ (sense) and 5′-CAGTAA TACGACTCACTATAGGGAG AAGGCTTAATCTA AAAAACTCCAACC AACCC-3′ (antisense); 5′-AGGA AGAGAGGATTTGGGTGT GAGAGAAGAGTTTT-3′ (sense) and 5′-CAG TAATACGACTCACTATAGGGA GAAGGCTAAAAACCCAA AAAAACCCCTTAAAC-3′ (antisense). The methylation data for individual units (1–5 CpG sites/unit) were analyzed using the Typer 4.0 software from Sequenom.

## SUPPLEMENTARY MATERIALS FIGURES AND TABLE



## References

[1] Torre LA, Bray F, Siegel RL, Ferlay J, Lortet-Tieulent J, Jemal A (2015). Global cancer statistics, 2012. CA Cancer J Clin.

[2] Ettinger DS, Akerley W, Borghaei H, Chang AC, Cheney RT, Chirieac LR, D'Amico TA, Demmy TL, Ganti AK, Govindan R, Grannis FW, Horn L, Jahan TM (2012). Non-small cell lung cancer. J Natl Compr Canc Netw.

[3] Samad TA, Srinivasan A, Karchewski LA, Jeong SJ, Campagna JA, Ji RR, Fabrizio DA, Zhang Y, Lin HY, Bell E, Woolf CJ (2004). DRAGON: a member of the repulsive guidance molecule-related family of neuronal- and muscle-expressed membrane proteins is regulated by DRG11 and has neuronal adhesive properties. J Neurosci.

[4] Liu X, Hashimoto M, Horii H, Yamaguchi A, Naito K, Yamashita T (2009). Repulsive guidance molecule b inhibits neurite growth and is increased after spinal cord injury. Biochem Biophys Res Commun.

[5] Samad TA, Rebbapragada A, Bell E, Zhang Y, Sidis Y, Jeong SJ, Campagna JA, Perusini S, Fabrizio DA, Schneyer AL, Lin HY, Brivanlou AH, Attisano L (2005). DRAGON, a bone morphogenetic protein co-receptor. J Biol Chem.

[6] Ye L, Lewis-Russell JM, Kyanaston HG, Jiang WG (2007). Bone morphogenetic proteins and their receptor signaling in prostate cancer. Histol Histopathol.

[7] Ye L, Mason MD, Jiang WG (2011). Bone morphogenetic protein and bone metastasis, implication and therapeutic potential. Front Biosci (Landmark Ed).

[8] Buijs JT, Rentsch CA, van der Horst G, van Overveld PG, Wetterwald A, Schwaninger R, Henriquez NV, Ten Dijke P, Borovecki F, Markwalder R, Thalmann GN, Papapoulos SE, Pelger RC (2007). BMP7, a putative regulator of epithelial homeostasis in the human prostate, is a potent inhibitor of prostate cancer bone metastasis *in vivo*. Am J Pathol.

[9] Alarmo EL, Parssinen J, Ketolainen JM, Savinainen K, Karhu R, Kallioniemi A (2009). BMP7 influences proliferation, migration, and invasion of breast cancer cells. Cancer Lett.

[10] Alarmo EL, Korhonen T, Kuukasjarvi T, Huhtala H, Holli K, Kallioniemi A (2008). Bone morphogenetic protein 7 expression associates with bone metastasis in breast carcinomas. Ann Oncol.

[11] Takahashi M, Otsuka F, Miyoshi T, Otani H, Goto J, Yamashita M, Ogura T, Makino H, Doihara H (2008). Bone morphogenetic protein 6 (BMP6) and BMP7 inhibit estrogen-induced proliferation of breast cancer cells by suppressing p38 mitogen-activated protein kinase activation. J Endocrinol.

[12] Cassar L, Nicholls C, Pinto AR, Li H, Liu JP (2009). Bone morphogenetic protein-7 induces telomerase inhibition, telomere shortening, breast cancer cell senescence, and death via Smad3. FASEB J.

[13] Topalian SL, Hodi FS, Brahmer JR, Gettinger SN, Smith DC, McDermott DF, Powderly JD, Carvajal RD, Sosman JA, Atkins MB, Leming PD, Spigel DR, Antonia SJ (2012). Safety, activity, and immune correlates of anti-PD-1 antibody in cancer. N Engl J Med.

[14] Xiao Y, Yu S, Zhu B, Bedoret D, Bu X, Francisco LM, Hua P, Duke-Cohan JS, Umetsu DT, Sharpe AH, DeKruyff RH, Freeman GJ (2014). RGMb is a novel binding partner for PD-L2 and its engagement with PD-L2 promotes respiratory tolerance. J Exp Med.

[15] Li J, Ye L, Mansel RE, Jiang WG (2011). Potential prognostic value of repulsive guidance molecules in breast cancer. Anticancer Res.

[16] Fujii M, Takeda K, Imamura T, Aoki H, Sampath TK, Enomoto S, Kawabata M, Kato M, Ichijo H, Miyazono K (1999). Roles of bone morphogenetic protein type I receptors and Smad proteins in osteoblast and chondroblast differentiation. Mol Biol Cell.

[17] Deng H, Ravikumar TS, Yang WL (2007). Bone morphogenetic protein-4 inhibits heat-induced apoptosis by modulating MAPK pathways in human colon cancer HCT116 cells. Cancer Lett.

[18] Nohe A, Keating E, Knaus P, Petersen NO (2004). Signal transduction of bone morphogenetic protein receptors. Cell Signal.

[19] Sanders AJ, Ye L, Li J, Mason MD, Jiang WG (2014). Tumour angiogenesis and repulsive guidance molecule b: a role in HGF- and BMP-7-mediated angiogenesis. Int J Oncol.

[20] Lv Z, Yang D, Li J, Hu M, Luo M, Zhan X, Song P, Liu C, Bai H, Li B, Yang Y, Chen Y, Shi Q (2013). Bone morphogenetic protein 9 overexpression reduces osteosarcoma cell migration and invasion. Mol Cells.

[21] Gatza CE, Elderbroom JL, Oh SY, Starr MD, Nixon AB, Blobe GC (2014). The balance of cell surface and soluble type III TGF-beta receptor regulates BMP signaling in normal and cancerous mammary epithelial cells. Neoplasia.

[22] Brambilla E, Travis WD, Colby TV, Corrin B, Shimosato Y (2001). The new World Health Organization classification of lung tumours. Eur Respir J.

[23] Edge SB, Compton CC (2010). The American Joint Committee on Cancer: the 7th edition of the AJCC cancer staging manual and the future of TNM. Ann Surg Oncol.

[24] Parr C, Watkins G, Mansel RE, Jiang WG (2004). The hepatocyte growth factor regulatory factors in human breast cancer. Clin Cancer Res.

[25] Jiang WG, Ye L, Patel G, Harding KG (2010). Expression of WAVEs, the WASP (Wiskott-Aldrich syndrome protein) family of verprolin homologous proteins in human wound tissues and the biological influence on human keratinocytes. Wound Repair Regen.

[26] Bechara A, Nawabi H, Moret F, Yaron A, Weaver E, Bozon M, Abouzid K, Guan JL, Tessier-Lavigne M, Lemmon V, Castellani V (2008). FAK-MAPK-dependent adhesion disassembly downstream of L1 contributes to semaphorin3A-induced collapse. EMBO J.

[27] Miyazono K (2009). Transforming growth factor-beta signaling in epithelial-mesenchymal transition and progression of cancer. Proc Jpn Acad Ser B Phys Biol Sci.

[28] Liang YY, Brunicardi FC, Lin X (2009). Smad3 mediates immediate early induction of Id1 by TGF-beta. Cell Res.

[29] Song H, Guo B, Zhang J, Song C (2009). Transforming growth factor-beta suppressed Id-1 Expression in a smad3-dependent manner in LoVo cells. Anat Rec (Hoboken).

[30] Lopez-Rovira T, Chalaux E, Massague J, Rosa JL, Ventura F (2002). Direct binding of Smad1 and Smad4 to two distinct motifs mediates bone morphogenetic protein-specific transcriptional activation of Id1 gene. J Biol Chem.

[31] Wahdan-Alaswad RS, Song K, Krebs TL, Shola DT, Gomez JA, Matsuyama S, Danielpour D (2010). Insulin-like growth factor I suppresses bone morphogenetic protein signaling in prostate cancer cells by activating mTOR signaling. Cancer Res.

[32] Su Y, Zheng L, Wang Q, Bao J, Cai Z, Liu A (2010). The PI3K/Akt pathway upregulates Id1 and integrin alpha4 to enhance recruitment of human ovarian cancer endothelial progenitor cells. BMC Cancer.

[33] Lo AK, Dawson CW, Lo KW, Yu Y, Young LS (2010). Upregulation of Id1 by Epstein-Barr virus-encoded LMP1 confers resistance to TGFbeta-mediated growth inhibition. Mol Cancer.

[34] Bhattacharya R, Kowalski J, Larson AR, Brock M, Alani RM (2010). Id1 promotes tumor cell migration in nonsmall cell lung cancers. J Oncol.

[35] Conrad S, Genth H, Hofmann F, Just I, Skutella T (2007). Neogenin-RGMa signaling at the growth cone is bone morphogenetic protein-independent and involves RhoA, ROCK, and PKC. J Biol Chem.

[36] Jiang Y, Liu MT, Gershon MD (2003). Netrins and DCC in the guidance of migrating neural crest-derived cells in the developing bowel and pancreas. Dev Biol.

[37] Schaffar G, Taniguchi J, Brodbeck T, Meyer AH, Schmidt M, Yamashita T, Mueller BK (2008). LIM-only protein 4 interacts directly with the repulsive guidance molecule A receptor Neogenin. J Neurochem.

[38] Zhou Z, Xie J, Lee D, Liu Y, Jung J, Zhou L, Xiong S, Mei L, Xiong WC (2010). Neogenin regulation of BMP-induced canonical Smad signaling and endochondral bone formation. Dev Cell.

