# Supplementary material for: Repulsive guidance molecule B inhibits metastasis and is associated with decreased mortality in non-small cell lung cancer
Source: Oncotarget. 2016 Feb 17;7(13):15678–89. doi: 10.18632/oncotarget.7463 (PMC4941269; doi:10.18632/oncotarget.7463)
Supplement: Supplementary file 1 [file oncotarget-07-15678-s001.pdf]

Repulsive guidance molecule B inhibits metastasis and is associated with decreased mortality in non-small cell lung cancer

Supplementary Materials

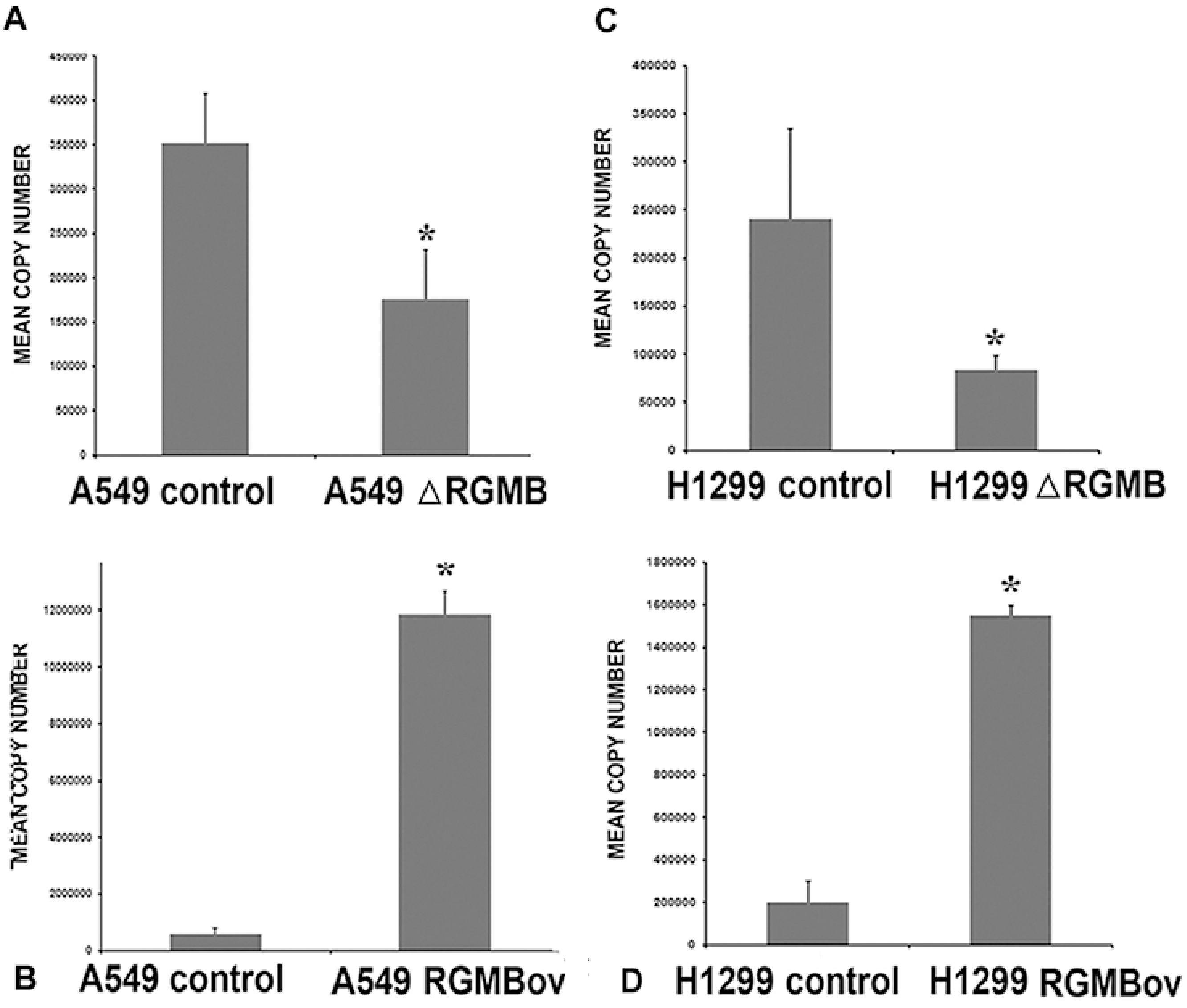

Supplementary Figure S1: Knockdown and overexpression of RGMB in A549 and H1299 cells.

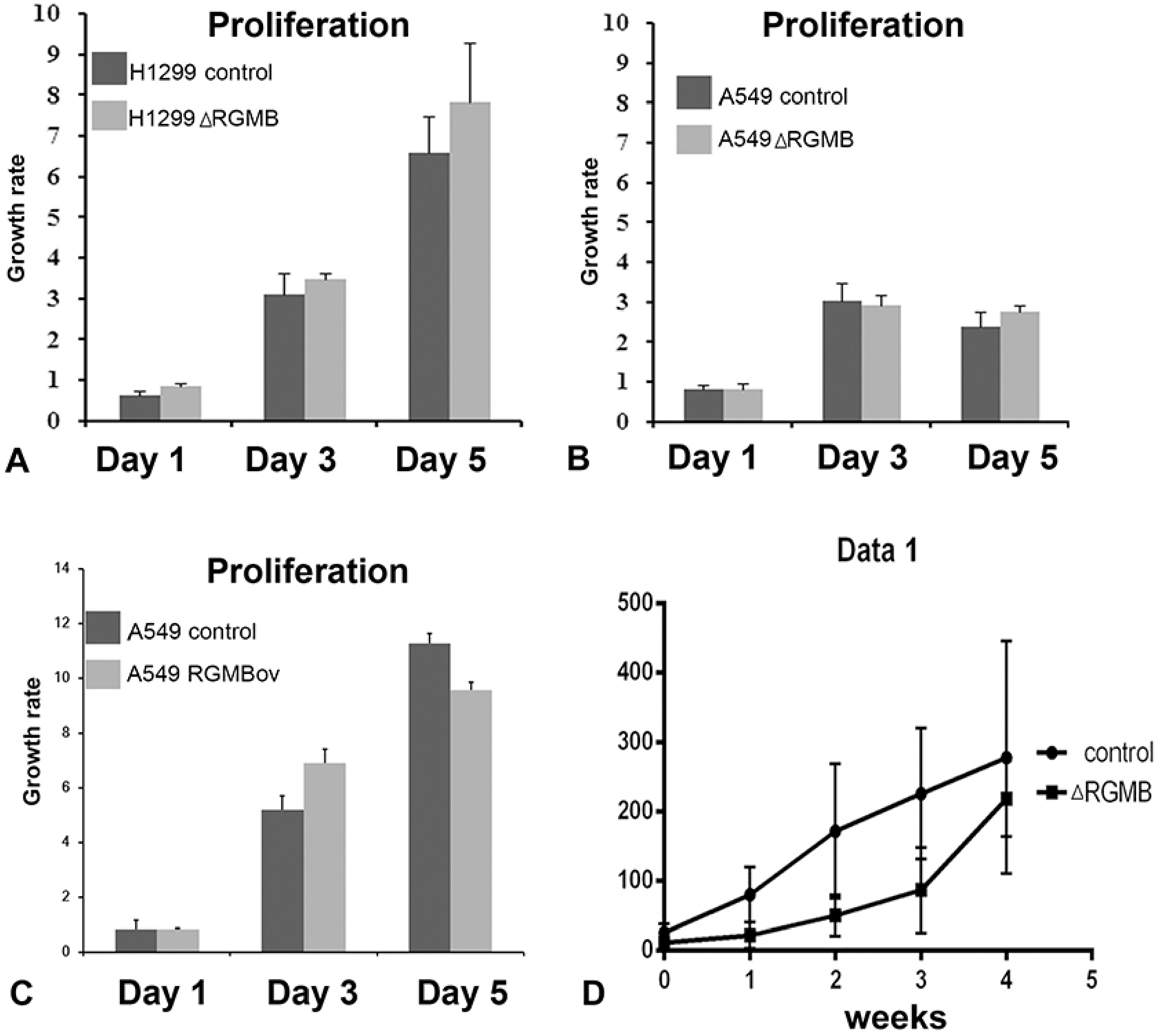

Supplementary Figure S2: Proliferation of A549 and H1299 RGMB knockdown and overexpression cells *in vitro* and *in vivo*.

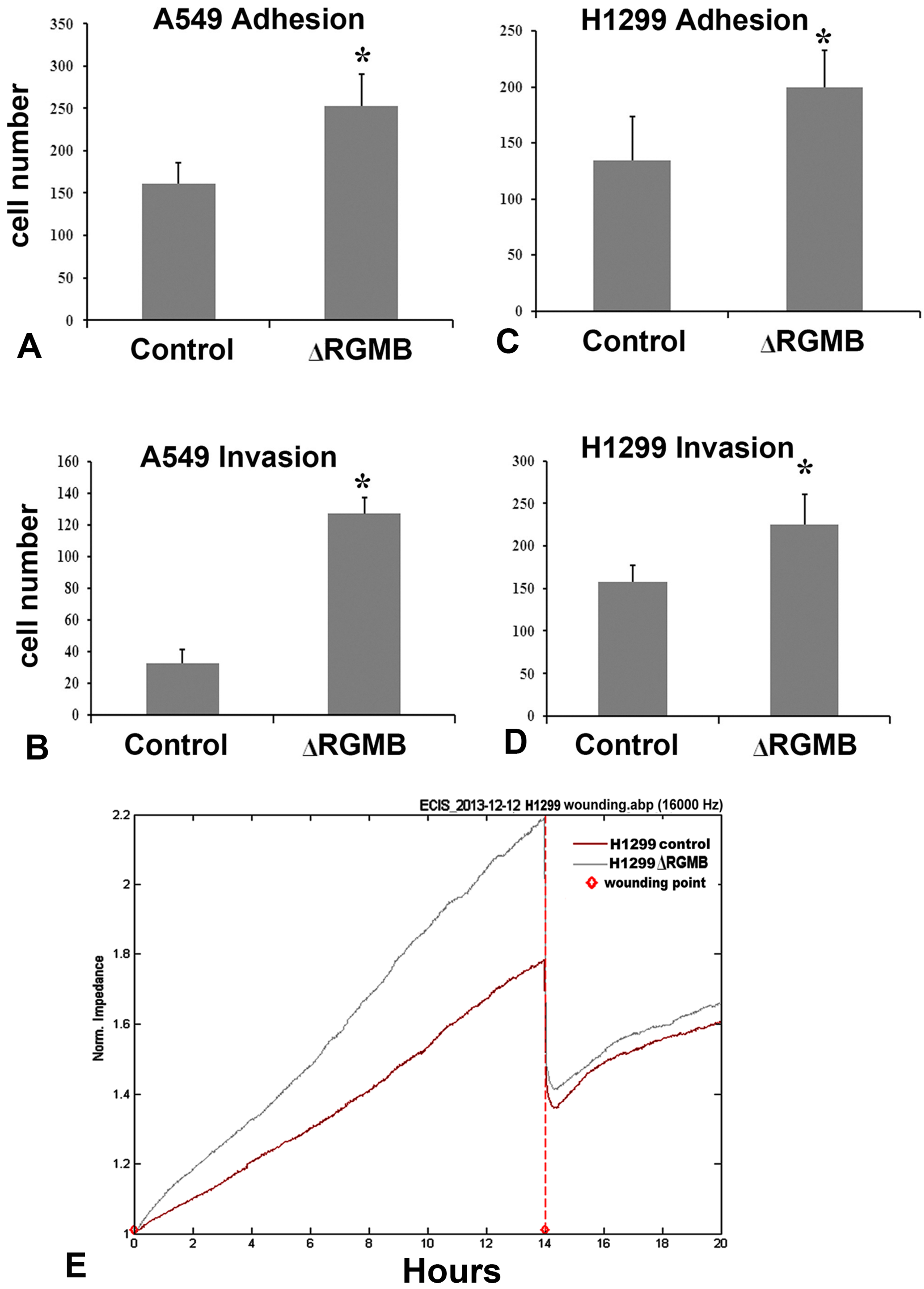

Supplementary Figure S3: Evaluation of RGMB knockdown A549 and H1299 cell adhesion, invasion, and ECIS-based migration.

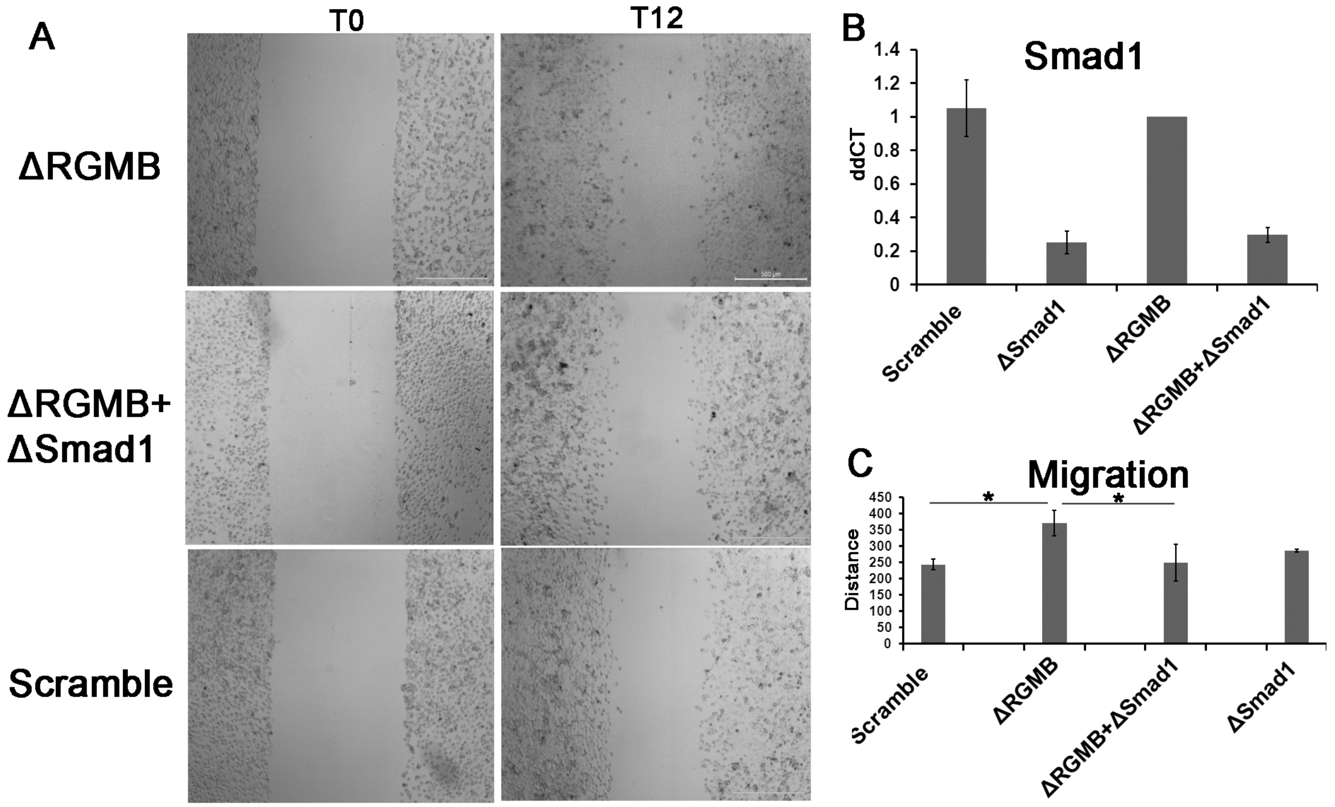

Supplementary Figure S4: Knockdown of smad1 diminishes the impact of RGMB suppression on migration after 12 hours.

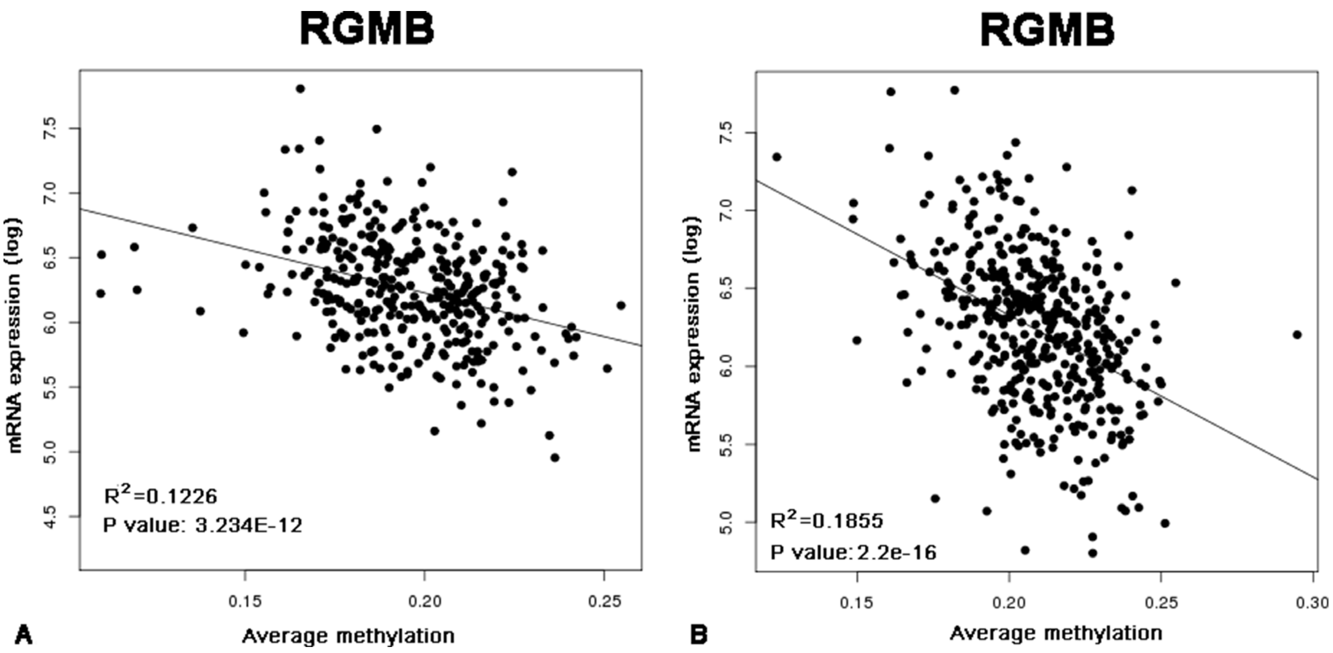

Supplementary Figure S5: The association between methylation and *RGMB* expression in LUSC (left) and LUAD (right).

Supplementary Table S1: Patient demographics and clinical characteristics

Characteristics		Discovery set		Validation set	
		NO.	%	NO.	%
Tissue type	Tumor	165	88.2	131	100
	Normal	22	11.8	0	0
Age (years)	≤ 65	113	60.4	96	73.3
	> 65	49	26.2	35	26.7
Gende	Female	64	34.2	58	44.3
	Male	101	54.0	73	55.7
Smoking status	Yes	58	31.0	31	23.7
	No	102	54.5	98	74.8
Histology	ADC+	139	74.3	131	100
	SCC+	17	9.1	0	0
	Other	9	4.8	0	0
Stage	I	53	28.3	61	46.6
	II	46	24.6	26	19.8
	IIIA	33	17.6	40	30.5
	IIIB	9	4.9	0	0
	IV	24	12.8	2	1.5
Differentiation	well	0	0	0	0
	moderate	117	62.6	99	75.6
	poor	38	20.3	32	24.4
Tumor nodularity	Unilateral pulmonary	158	84.5	126	96.2
	Bilateral pulmonary	7	3.7	5	3.8
Pleura involvement	Yes	88	47.1	94	71.8
	No	77	41.2	37	28.2
Lymph node involvement	Yes	92	49.2	52	39.7
	No	73	39.0	79	60.3
Vascular invasion	Yes	6	3.2	4	3.1
	No	159	85.0	127	96.9
Clinical outcome	Alive	109	58.3	109	83.2
	Death	54	28.9	20	15.3

+ADC: adenocarcinoma; SCC: squamous cell carcinoma.
